# Regular Meeting of the Fulton County Medical Society, January 20, 1916

**Published:** 1916-02

**Authors:** Allen H. Bunce


### REGULAR MEETING OF THE FULTON COUNTY MEDICAL SOCIETY, JANUARY 20, 1916.

(Reported by Allen H. Bunce, M. D.)

Dr. James E. Paullin presented the following cases:

(1) Aneurysm Ascending Arch of the Aorta.

(2) Aneurysm Transverse Arch of Aorta.

(3) Aneurysm Descending Arch of Aorta.

X-Ray Plate Illustrations of these cases were shown by Dr. Calloway. Dr. J. C. Patterson read a history of these cases for Dr. Paullin.

Dr. J. L. Campbell exhibited a case of Cerebellar Tumor upon which he has operated successfully. Dr. Campbell described the case as follows:

Mr. L. H. J. came under my care at Wesley Memorial Hospital December 19, 1915.

Diagnosis: Cerebellar Tumor, left lobe.

This diagnosis was based on the following facts:

Age 41. Occupation: Printer. When a small boy he struck the back of his head and was unconscious for about five hours. He has suffered more or less every day since with headaches—has been able to work at his trade until about three years ago, when he experienced difficulty in picking up the type he was attempting to set. He has had occasional attacks of vomiting, principally without nausea. The headaches grew worse and he has suffered with dizziness a great deal of the time. He staggers when he attempts to walk. If he attempts to stand with his head erect he falls backward and to the left. His sight has failed him, and finally gave out entirely, in the left eye. He became unable to distinguish faces twenty or thirty feet away.

Physical examination showed that he was only fairly nourished. His head and chest were bent forward when he attempted to stand. There is marked ataxia and staggering gait. He falls backward and to the left when unsupported. Also staggers to the left side when he attempts to walk without a cane. Choked disc marked. Reflexes normal or only slightly exaggerated.

An operation was determined on, and December 19th he was put to bed, given one ounce of castor oil at bed-time; soda bicarbonate 1 dram in water three times a day by mouth, and soda bicarbonate and glucose of each one dram in water (12 ounces) twice a day by rectum to be retained. The urine was negative, except few hyaline casts and 10 pus cells (in a voided specimen) to the two-thirds objective field.

December 22nd at 6:00 A. M. he was given a small S. S. enema. At 7:30 1 1-150 grain atropine and a high enema of whiskey one ounce, panopeptine, one ounce with normal saline solution 12 ounces, which was retained. At nine o'clock he was sent to the operating room with a temperature of 97. 4-5, pulse 100, respiration 18. Under ether anaesthesia he was placed as nearly as possible, with the arrangements at hand, on his abdomen. The head of the operating table was elevated about 10 inches above the level. A curved incision with the convexity upward, was made just above the superior curved line and entirely across the occiput. A second incision from the center of this was carried about 3 1-2 or four inches down the middle of the neck, and the sub-occipital space exposed. The bleeding was controlled above by a row of lock-stitched sutures, and below the larger vessels were clamped. The hemorrhage was considerable but not alarming. An elliptical opening was made in the left side of the occipital bone from just below the superior curved line almost to the margin of the foramen magnum and from the median line to the mastoid. The brain bulged some, but did not pulsate. The dura was opened about 1-4 of an inch below the upper margin of the opening in the skull and a hard rounded tumor was felt beneath the knee of the lateral sinus. It was firmly attached to the tentorium and loosely attached to the upper surface of the cerebellum. These adhesions were broken up and the tumor delivered. There was a considerable gush of blood and cerebro-spinal fluid. As soon as the tumor was delivered it stopped and the wound which up to this time had been oozing freely became dry. The sub-tentorial cavity was sponged out with gauze. The dura was closed with fine linen except a space just large enough to insert a small piece of rubber dam drainage. The wound was closed.

The operation consumed one hour and twenty-five minutes. The pulse which was 80 when the operation was begun rose to 126. Respiration which was 22 dropped to 16. While the tumor was being delivered he became livid. There was also some muscular twitching over the left side of the face. This stopped as soon as the intra-cranial manipulation was over. The respiration rose gradually to 24.

He made an uneventful recovery only vomiting one time while reacting, and required only three doses of morphine. The drainage was removed on the third day. The wound was completely healed at the end of two weeks. He has not been dizzy since the operation, and the headaches have entirely disappeared. He is now regaining his eyesight and can read the newspapers with considerable comfort.

Brain surgery, for the most part, is very discouraging. Failure is far more frequent than success. The tumor is not easily found, or cannot be removed when it is found. The operative mortality is great, ranging from 25 to 55%, except in Cushing's cases, where he reports 11 deaths in a series of 141 cases. Death unusually occurs within the first few hours after operation, largely due to the shock and loss of blood and disturbances of the respiratory centers.

In a most interesting article in the Journal of the American Medical Association, Volume LXIV, No. 3, Page 169, Cushing reviews results obtained in some of the large European Clinics and report his own. He has operated 36 times on 29 Cerebellar tumors with 5 deaths, and 113 times on 101 cases of tumors in other localities with 6 deaths. He states that the final results may be classed as follows:

(1) About 5% will be completely cured.

(2) 10% will be practically relieved of all symptoms and can perform the duties pertaining to their occupation with comparative comfort.

(3) Perhaps 50% or 60% of all cases can be benefited, that is, relieved of most of the graver symptoms of the disease than before operation.

(4) 10 to 15% will continue to have progressive symptoms and go on to an untimely end.

(5) 10 to 14% will die at the time of operation.

Mr. L. H. J. will, I believe, come under Cushing second classification, for I feel sure that he will be able to earn a living, but will never completely recover his sight, although it has improved wonderfully since the operation.

The tumor removed by Dr. Campbell was exhibited by Dr. John Funke. A pathological examination of the tumor by Dr. Funke showed it to be a fibroma which was probably derived from the tentorium.

Dr. Grady Cannon described the findings upon the examination of the eyes of Dr. Campbell's patient.

Dr. Stewart R. Roberts compared the general appearance and condition of the patient at the present time to his condition before operation as this same case was exhibited by Dr. Roberts in December when he stated that a diagnosis of cerebellar tumor had been made. The patient walks with much greater ease and has a very decided improvement in sight and is relieved entirely of the severe headaches which he had previous to the operation.

Dr. S. T. Harris exhibited four cases which had become cured of pulmonary tuberculosis. His treatment of these cases consisted of artificial pneumothorax. Dr. John S. Derr showed X-Ray plates illustrating these cases.

Dr. Floyd W. McRae exhibited a fibroid tumor of the uterus which had been mistaken for pregnancy four years ago at which time an operation was done on the patient by another surgeon. He states that externally the uterus looked exactly like it contained a six months pregnancy. The tumor consisted of a fibroid filling the entire cavity of the uterus. Dr. McRae also exhibited a safety-pin which he had removed from the stomach of a small child which had been in the stomach thirty-four days without giving any symptoms. The pin was located in the stomach by X-Ray pictures made by Dr. Geo. M. Niles.

Dr. John S. Derr presented the following cases:

(1) Two plates of the stomach.

(2) One plate showing an Ulcer of the Duodenum.

Dr. E. P. Merritt read a paper: "Clinical Report on the use of Diarsenol in five cases of Primary Syphilis with Illustrations of Results Obtained."

Dr. Merritt's paper was discussed by Drs. W. L. Champion, Geo. M. Niles, W. B. Emery and Stewart R. Roberts.

Dr. E. C. Thrash read a paper on "The pathology of Sphlilis—A Newer View."

Dr. Cosby Swanson read a paper on "The Differential Diagnosis of the Skin Manifestations in Pellagra from other Dermatoses."

Dr. Swanson's paper was discussed by Drs. G. C. Mizell and Geo. M. Niles.

---

### REGULAR MEETING OF THE FULTON COUNTY MEDICAL SOCIETY, FEBRUARY 3, 1916.

(Reported by Allen H. Bunce, M. D.)

